# Cardiometabolic consequences of maternal hyperandrogenemia in male offspring

**DOI:** 10.14814/phy2.14941

**Published:** 2021-07-20

**Authors:** Yvonne Zuchowski, Carolina Dalmasso, Noha M. Shawky, Jane F. Reckelhoff

**Affiliations:** ^1^ Department of Cell and Molecular Biology University of Mississippi Medical Center Jackson MS USA; ^2^ Department of Pharmacology and Nutritional Sciences University of Kentucky Lexington KY USA; ^3^ Mississippi Center of Excellence in Perinatal Research University of Mississippi Medical Center Jackson MS USA

**Keywords:** angiotensin II, body mass, cholesterol, polycystic ovary syndrome

## Abstract

Polycystic ovary syndrome (PCOS) in women is characterized by hyperandrogenemia, obesity, and oligo‐ or anovulation. In addition, women with PCOS are often obese, with insulin resistance, hyperlipidemia, and elevated blood pressure. The cardiometabolic consequences for the male offspring of maternal hyperandrogenemia are unclear. The present studies tested the hypothesis that male offspring of a rat model of PCOS would develop cardiometabolic disease as adults. Female Sprague–Dawley rats (hyperandrogenemic females (HAF)) were implanted with dihydrotestosterone or placebo pellets (controls) at 4 weeks of age, and were mated at 10–12 weeks and allowed to lactate their offspring after birth. Body weights in male HAF offspring were lower at birth than in controls until postnatal day 4, but body weights remained similar between male control and HAF offspring from 2 to 8 weeks of age. However, at 16 weeks of age, body weight was lower in HAF male offspring, but there were no differences in fat mass or lean mass factored for body weight in HAF males, compared to controls. Plasma total cholesterol and HDL and proteinuria were higher and nitrate/nitrite excretion was lower in male HAF offspring than in controls. Baseline blood pressure was similar between HAF male offspring and controls, but HAF offspring had an exaggerated pressor response to angiotensin II infusion. These data suggest that adult sons of PCOS mothers may be at increased risk of cardiometabolic disease.

## Graphical abstract

Adult male offspring of a rat model of polycystic ovary syndrome is born small compared to controls, and as adults have lower body weights, with higher cholesterol and proteinuria. Although the male PCOS offspring have similar blood pressure as controls, they have an exaggerated pressor response to angiotensin II. These data suggest that male offspring of PCOS women may develop cardiometabolic disease as adults or with aging.

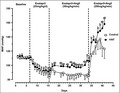

## 1 INTRODUCTION

Polycystic ovary syndrome (PCOS) is the most common endocrine pathology in women, affecting 5%‐10% of the population, often beginning in adolescence (Christakou & Diamanti‐Kandarakis, 2008; Diamanti‐Kandarakis, 2008; Escobar‐Morreale & San Millán, 2007; Witchel, 2006). PCOS is characterized by hyperandrogenemia, modest increases in blood pressure (BP), insulin resistance, and increased inflammation (Christakou & Diamanti‐Kandarakis, 2008; Diamanti‐Kandarakis, 2008; Escobar‐Morreale & San Millán, 2007; Witchel, 2006). The definitive criteria necessary for diagnosis of PCOS were determined in 2003, the Rotterdam Criteria (Azziz et al., 2006; Rotterdam ESHRE/ASRM‐Sponsored PCOS Consensus Workshop Group, 2004), and require the presence of two out of three characteristics, hyperandrogenemia, cystic ovaries, and oligo‐ or anovulation, for diagnosis.

PCOS women may have difficulty becoming pregnant and have a higher incidence of requiring assisted reproduction, such as in vitro fertilization (He et al., 2019; Kjerulff et al., 2011; Persson et al., 2019; Roos et al., 2011). For example, Persson et al., reported that Scandinavian PCOS women take longer to become pregnant and have fewer children than women without PCOS, but the probability of childbirth once pregnant was similar in PCOS women vs. controls (Persson et al., 2019). Other studies show that PCOS women may have a higher incidence of preeclampsia during pregnancy (Kjerulff et al., 2011; Roos et al., 2011). Children of PCOS women are often born small for gestational age, a condition called intrauterine growth restriction (IUGR) (Kjerulff et al., 2011; Roos et al., 2011; Sir‐Petermann et al., 2005).

While there are studies on pregnancy outcomes and offspring birth weights in women with PCOS (He et al., 2019; Kjerulff et al., 2011; Persson et al., 2019; Roos et al., 2011; Sir‐Petermann et al., 2005), there are no studies to our knowledge on the cardiovascular and metabolic consequences of hyperandrogenemia during pregnancy on the health of the offspring as adults or with aging. For example, de Wilde and colleagues studied children of PCOS women who were 2.5–4 years and 6–8 years of age (Wilde et al., 2018). Birth weights were not listed for these children nor were the data analyzed by sex, but the young PCOS offspring had significantly lower diastolic blood pressure (not measured by ambulatory monitoring, unfortunately), higher pulse pressure, higher left ventricular internal diameter, lower breast and abdominal circumference, but higher carotid intima‐media thickness (Wilde et al., 2018). In another study, Gunning et al. performed meta‐analyses of data from 298 PCOS offspring or controls from the Netherlands, Chile, and the United States (Gunning et al., 2020), some as old as 17 years. The male offspring of PCOS women had lower 2‐h fasting insulin, higher LDL‐cholesterol, and lower HDL‐cholesterol than did female PCOS offspring. Blood pressure and other cardiovascular parameters were not mentioned, however (Gunning et al., 2020). Thus there is a need to study the consequences of hyperandrogenemia during pregnancy on PCOS offspring.

In order to study the consequences of maternal hyperandrogenemia on body composition and cardiovascular complications later in life in male offspring, we use the hyperandrogenemic female (HAF) rat as a model (Dalmasso, Maranon, Patil, Bui, et al., 2016; Yanes et al., 2011). We showed previously that pregnancy occurs in approximately 60% of HAF rats, and that their offspring are born small for gestational age (Shawky et al., 2020). Unlike studies in animal models of PCOS and pregnancy in which androgens are given only in late pregnancy, the HAF dams are implanted with dihydrotestosterone pellets at 4–5 weeks of age such that the dams have elevated levels of androgens before, during and after their pregnancies, as PCOS women have (Falbo et al., 2010).

Thus in the present study using the HAF model, the hypothesis was tested that male offspring of HAF rats will be at increased risk for cardiovascular and metabolic abnormalities as adults.

## 2 METHODS

### 2.1 Hyperandrogenemic female (HAF) model and offspring generation

Female Sprague–Dawley (SD) rats were obtained at 3 weeks of age (Envigo), and maintained on standard chow (Teklad #8640) and tap water in a temperature‐controlled environment with 12 h: 12 h light:dark cycle. Females were implanted with dihydrotestosterone pellets (7.5 mg/90 day, s.c.; Innovative Research) or placebo pellets at 4 weeks of age to generate HAF or control females, respectively. Pellets were replaced every 85 days throughout their lives, as previously described (Dalmasso, Maranon, Patil, Bui, et al., 2016; Shawky et al., 2020; Yanes et al., 2011). All protocols followed the ARRIVE Guidelines and were reviewed by the Institutional Animal Care and Use Committee of the University of Mississippi Medical Center, and complied with the *Guidelines for the Care and Use of Laboratory Animals* by the National Institutes of Health (National Research Council (US) Committee for the Update of the Guide for the Care and Use of Laboratory Animals, 2011).

At 10 weeks of age, HAF and control rats were paired with male SD rats to induce pregnancy. Pregnancy occurred typically between 10 and 14 weeks of age, thus pellets were re‐implanted during pregnancy/lactation in all rats (16 weeks of age). Pups were weighed within 12 h of birth (Shawky et al., 2020). The numbers of pups per litter were similar between control and HAF dams, and the offspring body weights were lower for both male and female HAF offspring compared to those in control offspring (Shawky et al., 2020). Both control and HAF offspring were culled at 48 h after birth to 8 pups/litter (4 males and 4 females). Male and female HAF offspring and their respective controls were weaned at 21 days of age, and only male offspring were studied after that time. Only one male per litter for control and HAF offspring were used per parameter studied. Body weights were measured at postnatal days (PND) 1, 2, 4, 14 and 21, and then weekly after weaning until 8 weeks of age. Rats were studied at 16 weeks of age.

### 2.2 Body composition

At 16 weeks of age, body weight and composition, including fat and lean masses, were measured (during the morning from 9 AM‐12 noon), in male HAF and control offspring by EchoMRI (4 in 1‐900 model Body Composition Analyzer, EchoMRI LLC), as previously described (Dalmasso, Maranon, Patil, Bui, et al., 2016; Shawky et al., 2020). Data are presented as body weight (grams), fat or lean mass (grams), and fat or lean mass factored for body weight.

### 2.3 Urinary protein and nitrate/nitrite excretion

At 16 weeks of age, male HAF offspring and controls were placed in metabolic cages with free access to water, but no food to prevent both protein and nitrates from food to enter the urine. Urine was collected for 24 h, and protein excretion was measured by Bradford assay, using a commercially available reagent (Bio‐Rad), as we previously described (Dalmasso, Maranon, Patil, Bui, et al., 2016; Yanes et al., 2011). Data are presented as mg protein excreted per day. Urinary nitrate/nitrite excretion was also measured in the urine using Griess reagent and E.coli, as we previously described (Reckelhoff et al., 1998). Data are presented as μmol nitrate/nitrite excreted/d/kg body weight.

### 2.4 Measurement of metabolic parameters

Blood samples were collected from the retro‐orbital plexuses from HAF offspring and their respective controls after a 5 h fast (8 am–1 pm). Blood was centrifuged and plasma was collected in EDTA tubes. Plasma insulin was measured by ELISA (Crystal Chem, Elk Grove Village, IL, #90060), according to the manufacturer's recommendations. Plasma lipid profiles (total cholesterol (TC), triglycerides (TG), low‐density lipoprotein cholesterol (LDL‐C), and high‐density lipoprotein cholesterol (HDL‐C)) were measured by VET AXCEL chemistry analyzer by the Analytical and Assay Core at UMMC.

### 2.5 Telemetry implantation

Separate groups of HAF male offspring, 16 weeks of age, and their respective controls were implanted with radiotelemetry transmitters (HD‐S10, Data Sciences International) in the abdominal aorta below the renal arteries using aseptic technique, as previously described by us (Dalmasso, Maranon, Patil, Bui, et al., 2016; Sartori‐Valinotti et al., 2008; Shawky et al., 2020; Yanes et al., 2011). Rats were placed in cages above a receiver (RLA‐3000) and allowed 2 weeks of recovery prior to baseline mean arterial blood pressure (MAP) measurements. MAP was measured continuously, 24 h per day, in freely moving, conscious animals, using Ponemah 6.12 software (Data Science International).

Following measurement of baseline MAP for 5 days, rats were given an angiotensin‐converting enzyme inhibitor (enalapril, 25 mg/kg/day) in their drinking water to block endogenous angiotensin II (Ang II) synthesis, as we previously described (Sartori‐Valinotti et al., 2008). After 8 days of enalapril, HAF offspring and their controls were implanted with miniosmotic pumps (cat # 1002, Alzet) to deliver Ang II (50 ng/kg/min in saline), and MAP was recorded for 20 days. Then minipumps were replaced to deliver a higher dose of Ang II (200 ng/kg/min in saline), and MAP was recorded for an additional 7 days. Water intake was measured daily throughout the experiment to maintain stable enalapril dosing.

### 2.6 Statistical analyses

All data are expressed as means ±SEM. Comparisons between groups were analyzed by Student's *t*‐test (for 2 groups) or repeat measures ANOVA (for telemetry), as noted. Values of *p* ≤ 0.05 were considered statistically significant. Statistical analyses were performed using GraphPad Prism software (GraphPad Software Inc., V6.0c).

## 3 RESULTS

### 3.1 Characteristics of offspring

**FIGURE 1:**
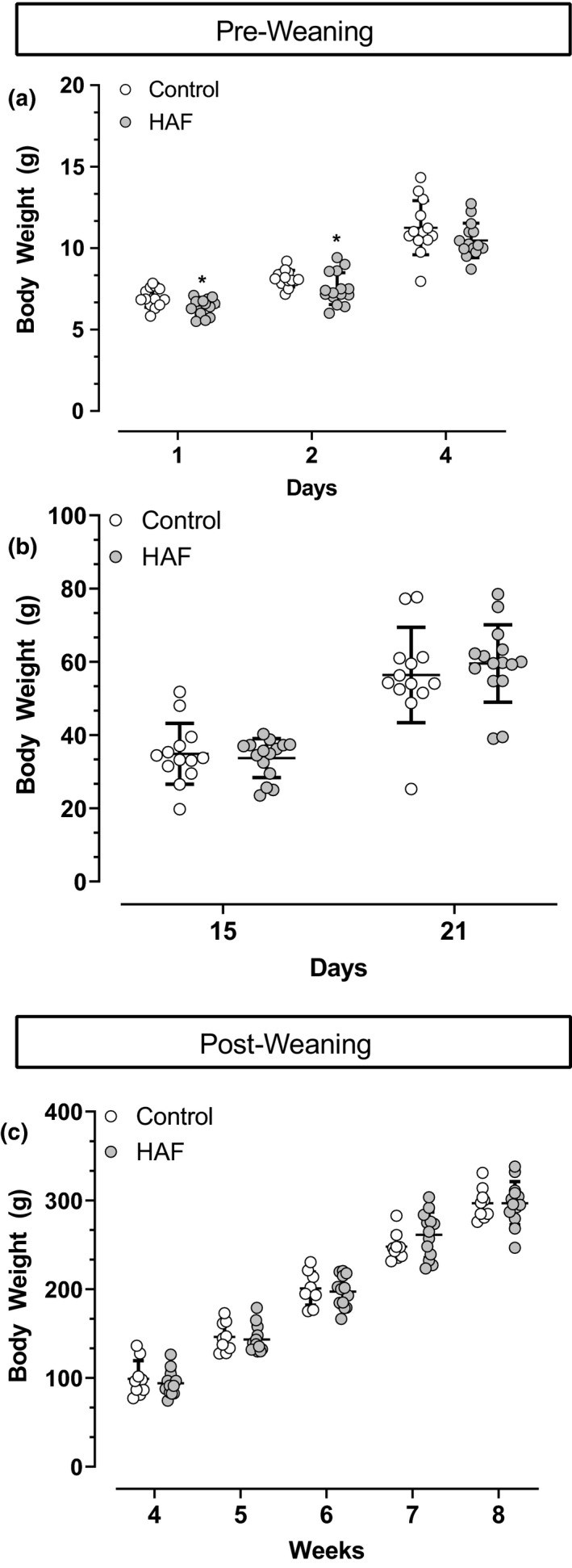
Body weights in male control (n = 13) and HAF offspring (n = 15) at postnatal day 1–4 (a), 15 and 21 (b) and at 4–8 weeks (c) of age. Statistical analyses were by Student's *t*‐test. Data are shown as standard deviation. *p < 0.05 compared with control male offspring

As mentioned above, we have shown previously that the numbers of pups per litter were similar between control and HAF offspring, but the birth weights of male and female HAF offspring were lower than those for control offspring (Shawky et al., 2020). As shown in Figure 1A, body weights remained lower in HAF males until PND 4, when they became similar to body weight in controls, and remained similar up to 8 weeks of age (Figure 1b,c).

As shown in Table 1, at 16 weeks of age, body weight was significantly lower in male HAF offspring than control males, but fat mass and fat mass factored for body weight were not statistically significantly different between groups. Lean mass was significantly lower in male HAF offspring compared to controls, but was not statistically significantly different between the groups when factored for body weight. As also shown in Table 1, urinary protein excretion was higher and nitrate/nitrite excretion was lower in male HAF offspring compared to those in control male offspring.

**TABLE 1:** Body weight and composition, protein and nitrate/nitrite excretion in male control and HAF offspring at 16 weeks of age

	Control offspring	HAF offspring
Body weight (g) (n = 11–17/group)	445.1 ± 8.7	422.6 ± 7.9*
Fat mass (g) (n = 11–17/group)	37.8 ± 3.3	33.2 ± 1.4
Fat mass/body weight (n = 11–17/group)	0.084 ± 0.006	0.078 ± 0.003
Lean mass (g) (n = 11–17/group)	385.4 ± 4.5	368.5 ± 6.9*
Lean mass/body weight (n = 11–17/group)	0.866 ± 0.009	0.872 ± 0.004
Urinary protein excretion (mg/d) (n = 12–19/group)	15.3 ± 1.3	25.5 ± 3.6*
Urinary nitrate/nitrite excretion (μmol/d/kg BW) (n = 14–18/group)	4.3 ± 0.4	4.8 ± 0.3

**Key:** HAF, offspring of hyperandrogenemic female dam. Data are expressed as mean ± SEM. Differences between groups were determined by Student's *t*‐test.

* *p* < 0.05, control vs. HAF.

As shown in Table 2, total cholesterol and HDL‐cholesterol were significantly higher in plasma from male HAF offspring compared to those in controls, but triglycerides, LDL‐cholesterol, insulin, and fasting blood glucose were similar between the groups.

**TABLE 2:** Lipids, insulin, and glucose in male control and HAF offspring

	Control offspring	HAF offspring
Total cholesterol (mg/dL) (n = 4–8)	98 ± 13	121 ± 5*
HDL‐cholesterol (mg/dL) (n = 4–8)	30 ± 3	38 ± 2*
LDL‐cholesterol (mg/dL) (n = 4–8)	16 ± 3	18 ± 1
Triglycerides (ng/dL) (n = 4–8)	110 ± 10	149 ± 2
Insulin (ng/ml) (n = 7–14)	1.0 ± 0.13	0.97 ± 0.10
Fasting glucose (mg/dL) (n = 4–7)	85 ± 2	85 ± 2

**Key:** HAF, offspring of hyperandrogenemic female dam. Data are expressed as mean **±SEM**. Statistical analyses by Student's *t*‐test.

* *p* < 0.05, compared to control offspring.

### 3.2 Pressor responses to ANG II

**FIGURE 2:**
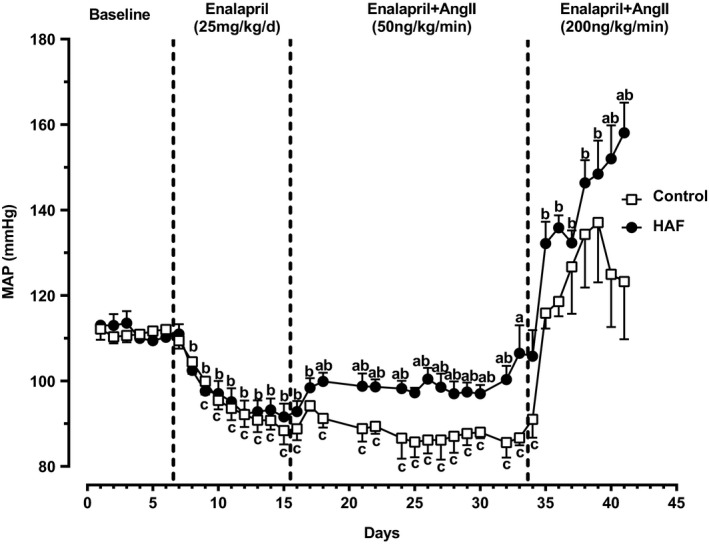
Baseline mean arterial pressure (MAP), depressor response to enalapril, and pressor response to Ang II (50 ng/kg/min or 200 ng/kg/min) in male control (n = 3) and HAF offspring (n = 5). MAP was measured by radiotelemetry. Statistical analyses were done by repeated measures ANOVA. Data are shown as standard error of the mean. ^a^ *p* < 0.05, MAP in male HAF offspring compared with control offspring; ^b^ *p* < 0.05, HAF offspring compared to baseline; ^c^ *p* < 0.05, control offspring compared to baseline

As shown in Figure 2, baseline MAP was not statistically significantly different between HAF and control offspring. Enalapril reduced MAP to similar levels in both groups. Low dose Ang II (50 ng/mg/min) for 20 days had no statistically significant effect on MAP in control offspring, but significantly increased MAP in HAF offspring although not above baseline levels. Higher dose Ang II (200 ng/kg/min) increased MAP in both control and HAF male offspring, but to a higher level in HAF offspring that was above baseline levels.

## 4 DISCUSSION

The present studies show that male HAF offspring have increased dyslipidemia, proteinuria and sensitivity to Ang II infusion compared to control offspring groups. These studies suggest that HAF male offspring may be at an increased risk for cardiometabolic disease with further aging.

We have shown previously that at 12–16 weeks of age, HAF rats (prior to pregnancy) have increased body weight and peri‐renal fat mass along with elevated plasma insulin, glucose, and cholesterol compared with control females (Yanes et al., 2011), similar to PCOS women (Christakou & Diamanti‐Kandarakis, 2008; Diamanti‐Kandarakis, 2008; Escobar‐Morreale & San Millán, 2007). HAF rats have elevated blood pressure prior to pregnancy (Yanes et al., 2011), as has also be reported in PCOS women (Christakou & Diamanti‐Kandarakis, 2008). We have also shown that the higher body weights prepregnancy in HAF rats remain so 48 h postpartum, but comparisons of percentage weight gains per body weight from pre to post‐pregnancy are similar between the control and HAF dams (Shawky et al., 2020). The numbers of pups per litter were also similar between HAF and control pregnancies (Shawky et al., 2020). Thus the mechanism(s) by which the HAF male offspring in the present study are born small for gestational age are not clear, nor is it clear why they develop catch up growth within 4 days that is continued through 8 weeks of age, but were then found to have lower body weight at 16 weeks of age. A potential confounder for these studies is that the DHT or placebo pellets were re‐implanted during pregnancy/lactation and this could have provided additional stress on the offspring. However, despite slightly lower body weights in HAF male offspring, neither fat mass nor lean mass per body weight ratios were different between the offspring groups at 16 weeks of age. In contrast, in the present study we found that their adult male HAF offspring, aged 16 weeks, had lower body weight, no differences in lean or fat mass when factored for body weight, no differences in insulin or glucose. However, the male HAF offspring did have elevated total cholesterol compared to that in control male offspring, although the mechanism is not clear. The HAF male offspring also were not hypertensive at baseline, but had an exaggerated pressor response to Ang II. Importantly, despite the fact that the male HAF offspring were normotensive, they had significantly higher protein excretion, suggesting the presence of renal injury that had not affected blood pressure as yet. In addition, since baseline blood pressures were not different, it is not likely that the level of proteinuria was indicative of deficits in renal hemodynamics either.

The mechanisms by which male HAF offspring may have an exaggerated response to Ang II, even at slow pressor doses (50 ng/kg/min), is not clear from the present studies. We have shown previously that the hypertension in virgin HAF rats stems from activation of the sympathetic nervous system (Palomba et al., 2015) and increased 20‐HETE in the renal microvasculature (Maranon et al.,). The mechanisms responsible for the Ang II pressor responses in male HAF offspring are not likely to include activation of the sympathetic nervous system since the male offspring are not obese as their mothers are (Falbo et al., 2010; Yanes et al., 2011). However, 20‐HETE could play a role in mediating the Ang II pressor response in the HAF offspring since 20‐HETE is upregulated in response to Ang II (Dalmasso, Maranon, Patil, Moulana, et al., 2016). It is also possible that some of the systems in place to offset an increase in blood pressure with Ang II are not present or are inactive in the male HAF offspring, such as the vasodilatory arm of the renin‐angiotensin system (Santos et al., 2019). In addition, the lower nitrate/nitrite excretion, an index of total body nitric oxide, in the HAF male offspring also suggests they may have increased oxidative stress that in the presence of Ang II could significantly increase their blood pressure (Reckelhoff & Romero, 2003). It is also possible that the HAF male offspring may have an abnormal upregulation of the endothelin system in response to Ang II that could play a role (Alexander et al., 2001). Endothelin can also increase oxidative stress (Sedeek et al., 2003). Thus future studies will be necessary to determine the mechanisms for the exaggerated Ang II response in the male HAF offspring.

The role that intrauterine growth restriction or low birth weight may play in mediating the cardiometabolic phenotype in male HAF rats is also not clear. Barker and colleagues reported that low birth weight, as a result of placental insufficiency, was associated with early mortality from cardiovascular disease (Barker et al., 1989). In support of this contention, in a model of reduced uterine perfusion pressure (RUPP) in rats, Alexander reported that low birth weight was associated with increased blood pressure in male offspring (Alexander, 2003), unlike in our male HAF offspring that had similar MAP as control offspring. Also, in contrast to our present studies, the body weights in the male RUPP offspring did not catch up to controls up to 12 weeks of age (Alexander, 2003). While the body weights in our HAF male offspring became similar to body weights in male control offspring by postnatal day 4, the body weights at 16 weeks were significantly lower in HAF offspring than in controls. Alexander did not measure metabolic parameters or urinary protein excretion, but she did find no difference in glomerular filtration rate between the control male offspring and the RUPP male offspring (Alexander, 2003). Future studies will be necessary to determine the consequences of hyperandrogenemic pregnancy on renal function in HAF male offspring as adults since they are proteinuric, and it will also be necessary to determine changes in metabolic and cardiovascular function in male HAF offspring as they age as we may see differences in MAP with aging and further metabolic changes.

Currently, there are no studies on the cardiovascular health of adult male offspring of PCOS women. One reason for this is that the Rotterdam Criteria for diagnosis of PCOS has been in place for less than 20 years (Azziz et al., 2006; Rotterdam ESHRE/ASRM‐Sponsored PCOS Consensus Workshop Group, 2004), so there is no population of adult male children of PCOS women to study as yet (Azziz et al., 2006; Rotterdam ESHRE/ASRM‐Sponsored PCOS Consensus Workshop Group, 2004). As noted above, studies in younger children of PCOS women show they have greater carotid intima‐media thickness and differences in fasting insulin and lipid levels (Gunning et al., 2020; Wilde et al., 2018). Thus based on the current studies, it is possible that male children of PCOS women may have an increased risk of developing cardiovascular and metabolic disease as adults or with aging. Unfortunately, men may not know their mothers had PCOS. Thus future studies into the cardiovascular health of men whose mothers had PCOS are important to determine if, in fact, they have increased risk of cardiovascular disease, especially with advancing age.

Finally, there are also no studies on the cardiovascular‐metabolic consequences of maternal PCOS in adult female offspring. There are studies into whether PCOS female offspring develop a PCOS phenotype themselves (elevated androgens, cystic ovaries, obesity), but the data are controversial with some studies showing they do and others showing they do not (Legro et al., 2017; Torchen et al., 2019). Because these studies were focused only on reproductive issues, neither blood pressure nor other metabolic parameters were measured. Thus future studies will be necessary to determine the cardiovascular and metabolic consequences, not just the reproductive consequences, of maternal PCOS in female offspring.

## DISCLOSURES

The authors have nothing to disclose other than their grant funding.

## AUTHOR CONTRIBUTIONS

Ms. Zuchowsky performed the metabolic studies and assays, analyzed the data, made figures, edited the drafts, and approved the final draft of the manuscript. Dr. Dalmasso developed the concept, oversaw the offspring husbandry, analyzed the data, edited the drafts, and approved the final draft of the manuscript. Dr. Shawky implanted the telemetry transmitters and collected the telemetry data, analyzed data, made figures, oversaw the studies, edited the drafts, and approved the final draft of the manuscript. Dr. Reckelhoff developed the concept, designed the experiments, analyzed data, wrote the drafts, and approved the final draft of the manuscript.
